# SERCA2a ameliorates cardiomyocyte T-tubule remodeling via the calpain/JPH2 pathway to improve cardiac function in myocardial ischemia/reperfusion mice

**DOI:** 10.1038/s41598-021-81570-4

**Published:** 2021-01-21

**Authors:** Shuai Wang, You Zhou, Yuanyuan Luo, Rongsheng Kan, Jingwen Chen, Haochen Xuan, Chaofan Wang, Junhong Chen, Tongda Xu, Dongye Li

**Affiliations:** 1 Institute of Cardiovascular Disease Research, Xuzhou Medical University, 84 West Huaihai Road, Xuzhou, 221002 Jiangsu People’s Republic of China; 2 Department of Cardiology, The Affiliated Hospital of Xuzhou Medical University, 99 West Huaihai Road, Xuzhou, 221006 Jiangsu People’s Republic of China

**Keywords:** Cardiovascular diseases, Cell signalling

## Abstract

Transverse-tubules (T-tubules) play pivotal roles in Ca^2+^-induced, Ca^2+^ release and excitation–contraction coupling in cardiomyocytes. The purpose of this study was to uncover mechanisms where sarco/endoplasmic reticulum Ca^2+^ ATPase (SERCA2a) improved cardiac function through T-tubule regulation during myocardial ischemia/reperfusion (I/R). SERCA2a protein expression, cytoplasmic [Ca^2+^]_i_, calpain activity, junctophilin-2 (JPH2) protein expression and intracellular localization, cardiomyocyte T-tubules, contractility and calcium transients in single cardiomyocytes and in vivo cardiac functions were all examined after SERCA2a knockout and overexpression, and Calpain inhibitor PD150606 (PD) pretreatment, following myocardial I/R. This comprehensive approach was adopted to clarify SERCA2a mechanisms in improving cardiac function in mice. Calpain was activated during myocardial I/R, and led to the proteolytic cleavage of JPH2. This altered the T-tubule network, the contraction function/calcium transients in cardiomyocytes and in vivo cardiac functions. During myocardial I/R, PD pretreatment upregulated JPH2 expression and restored it to its intracellular location, repaired the T-tubule network, and contraction function/calcium transients of cardiomyocytes and cardiac functions in vivo. SERCA2a suppressed calpain activity via [Ca^2+^]_i_, and ameliorated these key indices. Our results suggest that SERCA2a ameliorates cardiomyocyte T-tubule remodeling via the calpain/JPH2 pathway, thereby improving cardiac function in myocardial I/R mice.

## Introduction

Worldwide, coronary heart disease (CHD) is the main cause of morbidity and mortality, and brings a heavy physical and psychological burden to patients and their families^1^. Several coronary revascularization strategies have been used to treat CHD, thereby improving blood perfusion of the ischemic myocardium. However, studies have demonstrated that reperfusion of the ischemic myocardium sometimes triggers no flow, reperfusion arrhythmia, and cardiac dysfunction, which are termed ischemia/reperfusion (I/R) injury^2,3^. At present, no effective treatment exists for myocardial I/R injury, therefore it is important to investigate pathological mechanisms and determine effective treatment targets.

During myocardial I/R injury, Ca^2+^ overload is an important injury mechanism, as during this pathological process, cardiomyocyte structure and function are damaged^3,4^. Ca^2+^ overload is primarily related to destruction of the Ca^2+^ cycle. During cardiac diastolic phase, cytoplasmic Ca^2+^ (approximately 70%) is retrieved into the sarcoplasmic reticulum (SR) via the action of sarco/endoplasmic reticulum Ca^2+^ ATPase (SERCA2a)^5,6^. Therefore, this protein plays a vital role in maintaining Ca^2+^ homeostasis in cardiomyocytes.

When we reviewed the literature, previous SERCA2a studies mainly focused on heart failure (HF). SERCA2a expression in heart failure tissue is down-regulated, and is associated with decreased myocardial contractility^7^. Transforming SERCA2a gene into HF animals can improve cardiac function^8,9^. However, few studies have focused on SERCA2a and myocardial I/R; an early study showed that myocardial I/R injury decreased SERCA2a expression^10^. Our previous studies showed that SERCA2a expression, activity and stability were decreased during myocardial I/R injury^11–13^. However, the effects of SERCA2a in maintaining Ca^2+^ homeostasis during myocardial I/R require further study.

Heinzel et al*.* demonstrated that T-tubule density was reduced in chronically ischemic myocardium, resulting in reduced Ca^2+^ release into the SR^14^. Lyon et al*.* observed that SERCA2a gene therapy for HF could repair T-tubule functional networks in mice cardiomyocytes^15^. Rapid and synchronized Ca^2+^ release occurs in whole cardiomyocytes after action potential generation, which is closely related to T-tubules. T-tubules are special tubular structures formed by cardiomyocyte membrane invaginations at the Z-line level of the sarcomere. The structure is rich in ion channels which are important for excitation–contraction coupling, resting membrane potential maintenance, signal transduction, action potential initiation and regulation^16^. L-type Ca^2+^ channels (LTCC) on T-tubules and Type 2 ryanodine receptors (RyR2)on SRs, form a conjunction membrane complex that is the structural basis of Ca^2+^-induced, Ca^2+^ release and excitation–contraction coupling in cardiomyocytes. However, the exact mechanism of SERCA2a regulation of T-tubule remodeling is unclear.

Several studies have demonstrated that the dyadic anchoring protein, junctophilin-2 (JPH2)^17–23^ is a key T-tubule regulator. JPH2 expression was reduced in myocardial I/R injury^24^, and the protein is the main junctophilin subtype in myocardium. It binds LTCC on T-tubules to RYR2 on SRs to form dyads. In HF animal models and HF patients, JPH2 expression was down-regulated and its intracellular localization was altered^17,19,25^. T-tubule maturation was abnormal after JPH2 knockout in neonatal mice, and T-tubules were damaged in adult mice cardiomyocytes or in vivo after JPH2 knockout^18,19^. Therefore, JPH2 plays a pivotal role in the formation and maintenance of T-tubules.

Wu et al*.* determined that JPH2 was the hydrolysis substrate of calpain, which is a family of widely expressed, intracellular cysteine proteases dependent on Ca^2+^^26^. Calpain cleaves a variety of proteins that maintain normal cardiac functions, including protein kinase C, calcineurin, caspase, SERCA2a, LTCC, JPH2, etc^27^. Calpain activity increases during several pathological processes, e.g. myocardial I/R, myocardial infarction and pressure overload^28,29^. Increased calpain activity cleaves JPH2 and destroys T-tubules, whereas calpain inhibition restores JPH2 expression and T-tubule structure and function^24^.

In this study, our objective was to verify the hypothesis that SERCA2a regulates cardiomyocyte T-tubule remodeling via the calpain/JPH2 pathway, thereby improving cardiac function. To do this, we used a SERCA2a knockout model, SERCA2a overexpression and PD150606 (PD) pretreatment in a myocardial I/R mouse model. We confirmed that calpain-mediated JPH2 proteolysis, and the corresponding restoration of T-tubule integrity are important mechanisms for SERCA2a-mediated improvements in cardiac function during myocardial I/R.

## Results

### The establishment of a mouse I/R injury model and the isolation of adult mice cardiomyocytes

**Figure 1:**
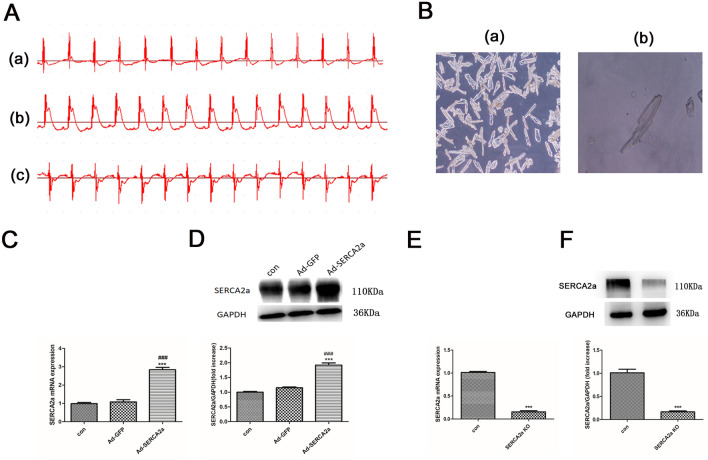
Electrocardiogram traces in myocardial I/R mice, isolated adult mice cardiomyocytes and the efficiency of SERCA2a overexpression and knockout. (**A**) Dynamic changes of electrocardiogram at different stages of myocardial I/R ((a) Basic state, (b) Ischemia state, (c) Reperfusion state). (**B**) Newly isolated adult mouse cardiomyocytes with different magnification ((a) 100 × , (b) 400 ×). (**C**, **E**) Expression of SERCA2a mRNA in each group. (**D**, **F**) The level of SERCA2a protein expression in each group. GAPDH was used as the internal control. The grouping of gels/blots cropped from different parts of the same gel. Uncropped blots are available in Supplementary Fig. 1D, F. Individual data points are presented in Supplemental Fig. 1C-F. ***P < 0.001 versus con; ###*P* < 0.001 versus Ad-GFP. n = 3–4; mean ± SEM.

To confirm the I/R mouse model, we recorded electrocardiograms. As shown (Fig. 1A), the ST segment was at baseline before myocardial ischemia, and was significantly elevated after myocardial ischemia, falling back to baseline after myocardial reperfusion. This indicated the successful establishment of the myocardial mouse I/R model.

To detect T-tubules, JPH2 localization, myocardial contraction and calcium transients, mice cardiomyocytes were isolated using the Langendorff system (Fig. 1B).

### SERCA2a overexpression and knockout

SERCA2a overexpression and knockout efficiencies are shown (Fig. 1C–F). Protein expression of SERCA2a and its mRNA were significantly upregulated in myocardial tissue when transfected by the SERCA2a overexpression adenovirus (all *P* < 0.001). When compared with the control group, the SERCA2a KO significantly reduced SERCA2a protein and mRNA expression (< 20%, *P* < 0.001).

### SERCA2a regulates calpain activity via [Ca^2+^]_i_

**Figure 2:**
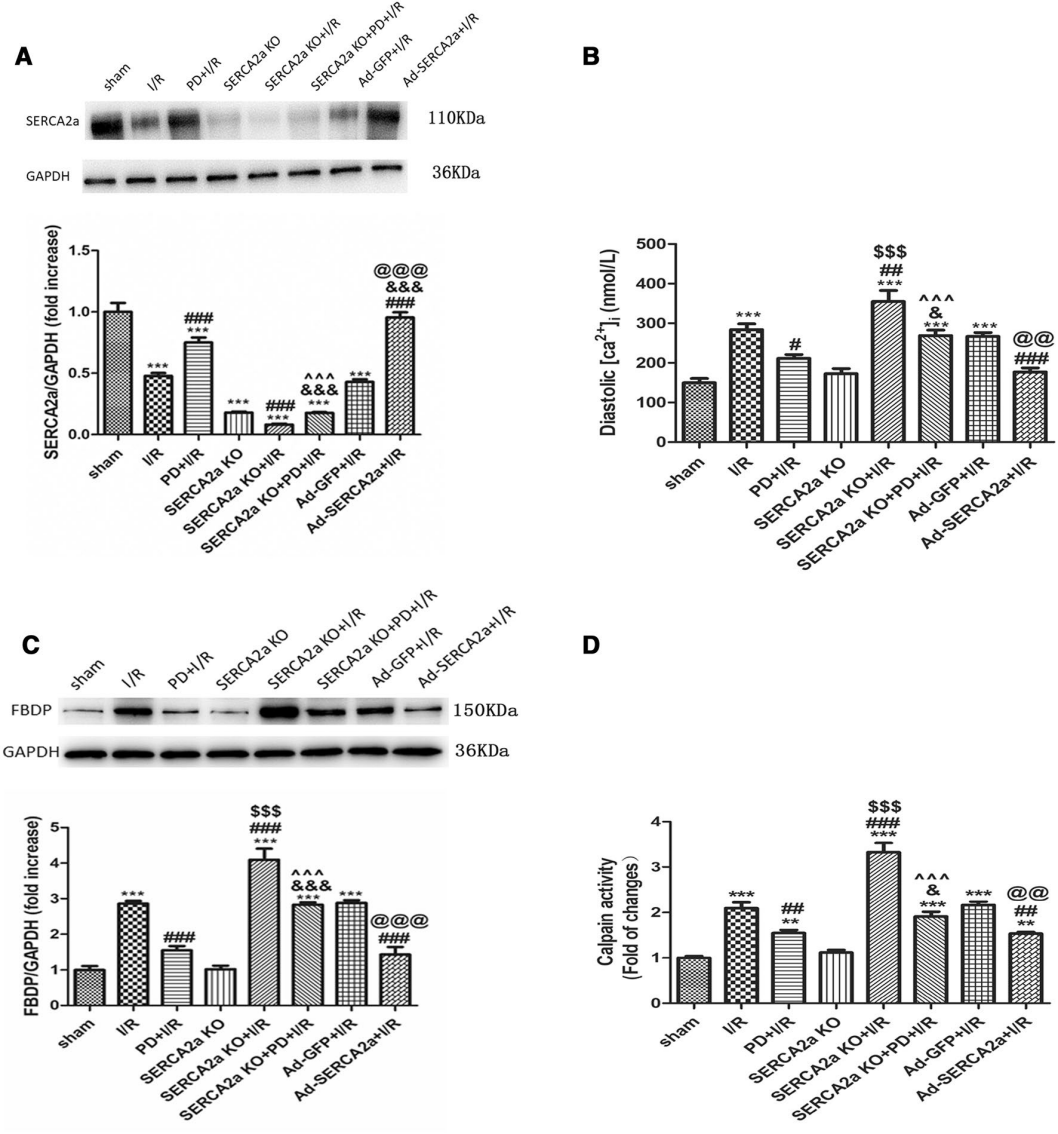
SERCA2a regulated calpain activity via [ca^2+^]_i_ in myocardial I/R mice. (**A**) The level of SERCA2a protein expression in each group. GAPDH was used as the internal control. The grouping of gels/blots cropped from different parts of the same gel. Uncropped blots are available in Supplementary Fig. 2A. (**B**) The diastolic [ca^2+^]_i_ of cardiomyocyte in each group. (**C**, **D**) The calpain activity in each group. GAPDH was used as the internal control. The grouping of gels/blots cropped from different parts of the same gel. Uncropped blots are available in Supplementary Fig. 2C. Individual data points are presented in Supplemental Fig. 2A-D. **P < 0.01, ***P < 0.001 versus sham; ^#^*P* < 0.05, ^##^*P* < 0.01, ^###^*P* < 0.001 versus I/R; ^&^*P* < 0.05, ^&&&^*P* < 0.001 versus PD + I/R; ^$$$^*P* < 0.001 versus SERCA2a KO; ^^^^^*P* < 0.001 versus SERCA2a KO + I/R; ^@@^*P* < 0.01, ^@@@^*P* < 0.001 versus Ad-GFP + I/R. n = 3; mean ± SEM.

I/R significantly decreased SERCA2a expression (*P* < 0.001) (Fig. 2A). When compared with the I/R group, PD pretreatment and SERCA2a overexpression partly reversed this expression (*P* < 0.001). However, SERCA2a KO + I/R further decreased SERCA2a expression, but there were no significant differences (*P* > 0.05). When compared with the PD + I/R group, SERCA2a expression in the SERCA2a KO + PD + I/R group was lower.

SERCA2a is vital for maintaining intracellular Ca^2+^ homeostasis, therefore we detected cytoplasmic [Ca^2+^]_i_ in different treatment groups. As shown (Fig. 2B), cytoplasmic [Ca^2+^]_i_ increased after I/R, however PD pretreatment and SERCA2a overexpression decreased [Ca^2+^]_i_. SERCA2a KO + I/R further aggravated Ca^2+^ overload. When compared with the PD + I/R group, [Ca^2+^]_i_ in SERCA2a KO + PD + I/R group was higher.

To verify the effects of SERCA2a on calpain activity via [Ca^2+^]_i_ in I/R, calpain activity was investigated. Activity was expressed as relative fluorescence, and FBDP expression levels as detected by western blotting. As shown (Fig. 2C-D), the trend in calpain activity was consistent with that of [Ca^2+^]_i_, suggesting that SERCA2a regulates calpain activation via [Ca^2+^]_i_ in myocardial I/R.

### SERCA2a regulates JPH2 expression and intracellular location, via calpain

**Figure 3:**
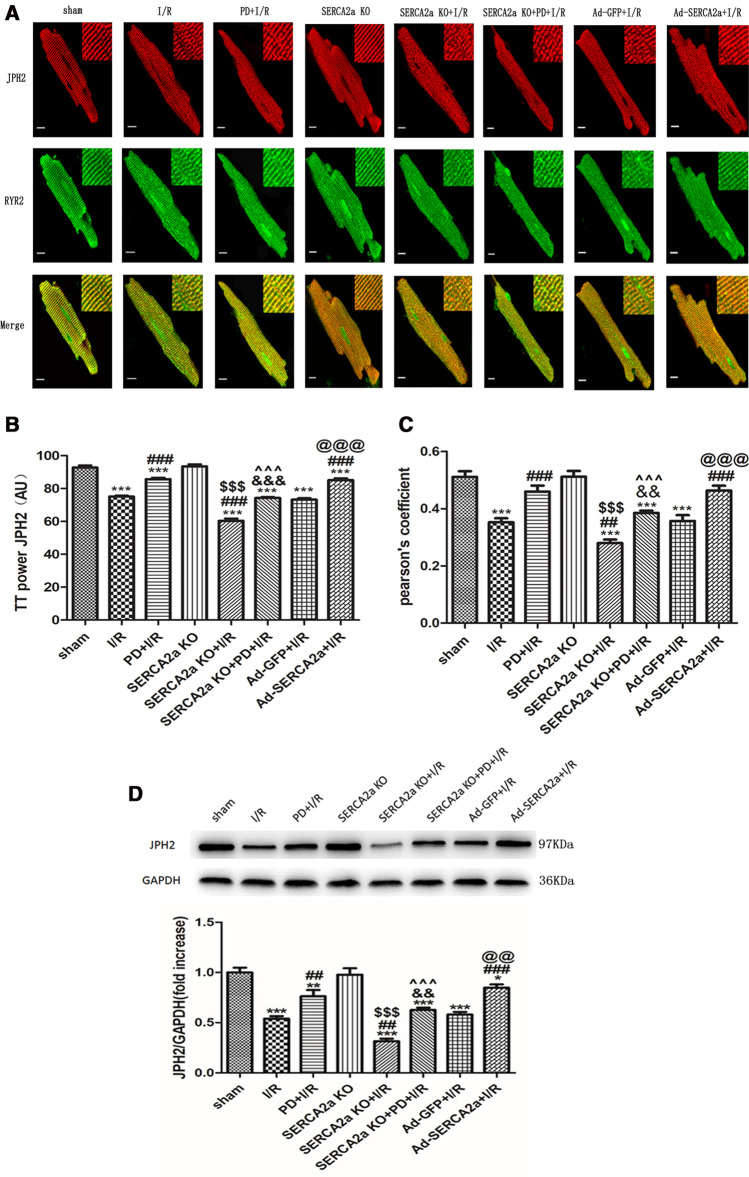
SERCA2a regulated the expression and location of JPH2 protein via calpain in myocardial I/R mice. (**A**) Immunofluorescence confocal microscopy images of cardiomyocytes in each group incubated with antibodies against JPH2 (read) and RyR2 (green), bar = 10 µm. (**B**) TTpower JPH2 as a measure of JPH2 spatial organization in each group. (**C**) Pearson’s coefficient for colocalization of JPH2 and RyR2 in each group. (**D**) Expression of JPH2 protein in each group. GAPDH was used as the internal control. The grouping of gels/blots cropped from different parts of the same gel. Uncropped blots are available in Supplementary Fig. 3D. Individual data points are presented in Supplemental Fig. 3B-D. *P < 0.05, **P < 0.01, ***P < 0.001 versus sham; ^##^*P* < 0.01, ^###^*P* < 0.001 versus I/R; ^&&^*P* < 0.01, ^&&&^*P* < 0.001 versus PD + I/R; ^$$$^*P* < 0.001 versus SERCA2a KO; ^^^^^*P* < 0.001 versus SERCA2a KO + I/R; ^@@^*P* < 0.01, ^@@@^*P* < 0.001 versus Ad-GFP + I/R. n = 18 to s31 cells from 3–5 hearts per group; mean ± SEM.

As shown (Fig. 3A–C), JPH2 in the sham group showed a regular striated pattern that overlapped with RYR2 in cardiomyocytes. TTpower JPH2 reflected the regularity of JPH2 protein distribution in cardiomyocytes, and Pearson's coefficients reflected the co-localization of JPH2 and RYR2. When compared with the sham group, TTpower JPH2 and Pearson's coefficients of cardiomyocytes decreased after I/R (*P* < 0.001). When pretreated with PD and SERCA2a overexpression before I/R, TTpower JPH2 and Pearson's coefficients increased (*P* < 0.001), suggesting that calpain and SERCA2a affected JPH2 location during I/R. There was no significant difference for TTpower JPH2 and Pearson's coefficients between SERCA2a KO and the sham group (*P* > 0.05), suggesting that SERCA2a knockout alone did not affect JPH2 location. However, when compared with the I/R group, TTpower JPH2 and Pearson's coefficients in the SERCA2a KO + I/R group were decreased (*P* < 0.001; *P* < 0.01), and were lower than those in the SERCA2a KO group (*P* < 0.001). These results suggested that SERCA2a may have affected JPH2 localization during I/R. In the SERCA2a KO + PD + I/R group, TTpower JPH2 and Pearson's coefficients were lower than the PD + I/R group (*P* < 0.001; *P* < 0.01), while they were higher than the SERCA2a KO + I/R group (*P* < 0.001). Similarly, as shown (Fig. 3D), the JPH2 expression trend was consistent with JPH2 localization, suggesting overall that SERCA2a regulated the localization and expression of JPH2 through calpain in myocardial I/R. As shown (Fig. S1), there was no significant difference between the sham and the SERCA2a overexpression group (P > 0.05). In addition, there was also no significant difference between the PD + SERCA2a overexpression and the SERCA2a overexpression group (P > 0.05).

### SERCA2a regulates T-tubule remodeling via calpain/JPH2

**Figure 4:**
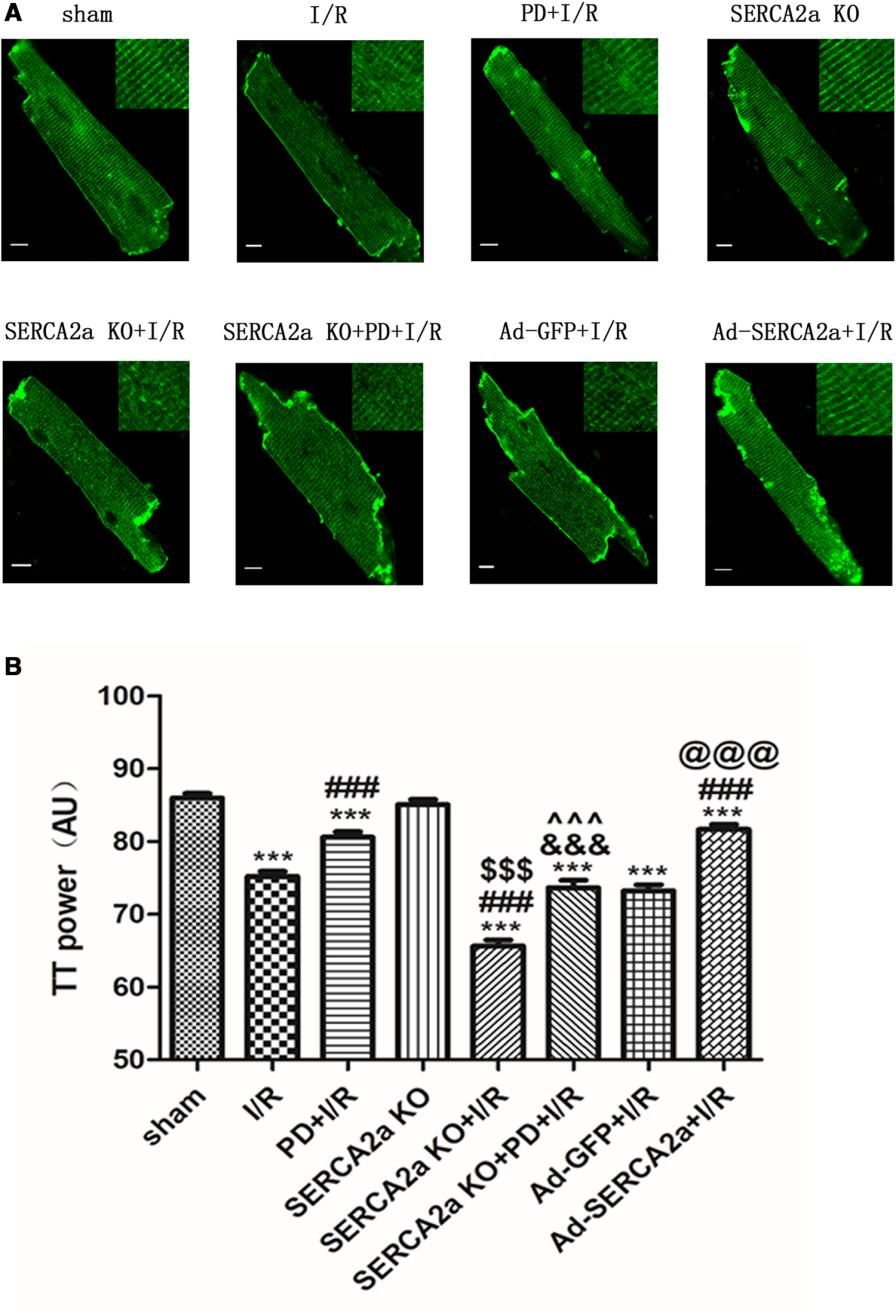
SERCA2a regulated T-tubule remodeling via calpain/JPH2 in myocardial I/R mice. (**A**) Confocal microscopy images of cardiomyocytes in each group stained with MM 4–64 (green), bar = 10 µm. (**B**) TTpower as a measure of T-tubule spatial organization in each group. Individual data points are presented in Supplemental Fig. 4B. ***P < 0.001 versus sham; ^###^*P* < 0.001 versus I/R; ^&&&^*P* < 0.001 versus PD + I/R; ^$$$^*P* < 0.001 versus SERCA2a KO; ^^^^^*P* < 0.001 versus SERCA2a KO + I/R; ^@@@^*P* < 0.001 versus Ad-GFP + I/R. n = 27 to 67 cells from 3–5 hearts per group; mean ± SEM.

JPH2 is an important regulator of T-tubules^17–23^. To verify SERCA2a regulation of T-tubule remodeling through calpain/JPH2 in I/R, we detected T-tubule changes under different intervention conditions. As shown (Fig. 4), cardiomyocyte T-tubules in the sham group were organized, showing striated patterns near the sarcomere Z lines, at regular 2 mm intervals. TTpower was used to reflect the integrity of T-tubules. When compared with the sham group, myocardial I/R destroyed T-tubules in cardiomyocytes (*P* < 0.001). After PD pretreatment, the TTpower of cardiomyocytes increased (*P* < 0.001), suggesting that calpain affected T-tubule structures during myocardial I/R. There was no statistically significance in TTpower between the SERCA2a KO group and the sham group (*P* > 0.05), suggesting that SERCA2a knockout alone did not destroy T-tubules, but SERCA2a KO further decreased TTpower in I/R (*P* < 0.001). Also, SERCA2a overexpression increased TTpower in I/R (*P* < 0.001).

These results indicated that SERCA2a affected T-tubule structures during I/R. The TTpower of cardiomyocytes in the SERCA2a KO + PD + I/R group was lower than the PD + I/R group (*P* < 0.001), but significantly higher than the SERCA2a KO + I/R group (*P* < 0.001). These results further suggested that SERCA2a regulates T-tubule remodeling by calpain in myocardial I/R.

### SERCA2a ameliorates contractility/calcium transient of cardiomyocytes and in vivo cardiac function via the calpain/JPH2 pathway

**Figure 5:**
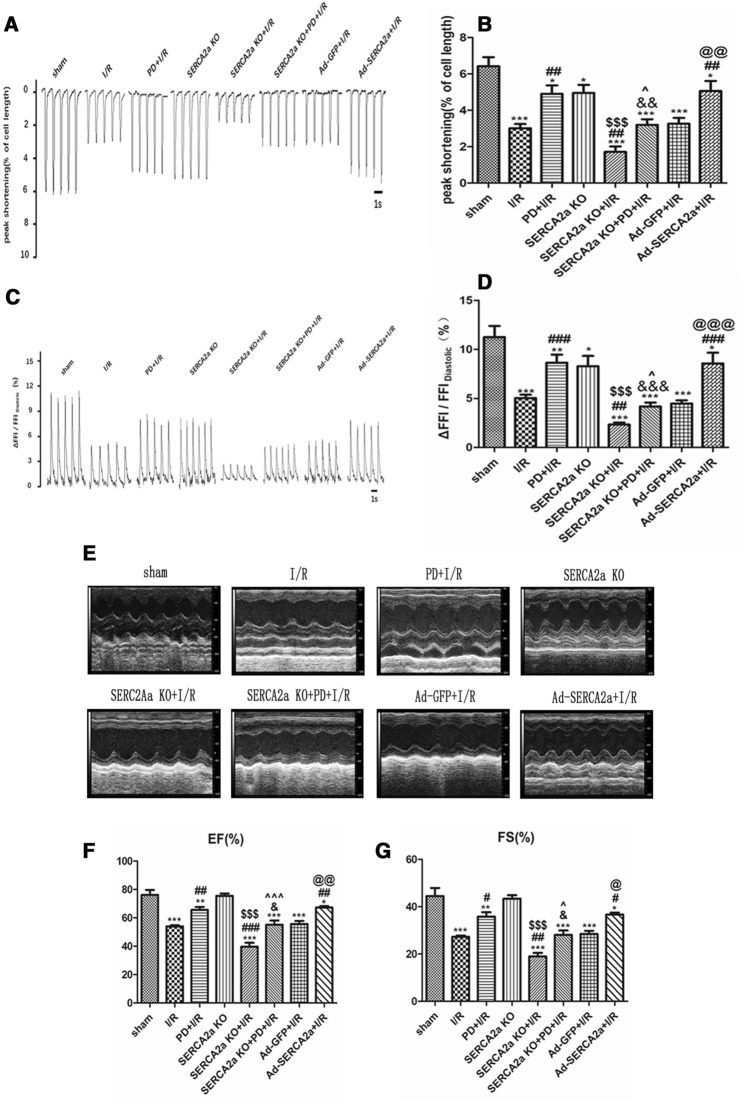
SERCA2a ameliorated contractility /calcium transient of cardiomyocytes and cardiac function in vivo via calpain/JPH2/T-tubule in myocardial I/R mice. (**A**) Representative contractility tracings in each group. n = 11 to 22 cells from 3–5 hearts per group; mean ± SEM. (**B**) Summary data of contractility in each group. (**C**) Representative Ca^2+^ transient tracings in each group. n = 11 to 22 cells from 3–5 hearts per group ; mean ± SEM. (D) Summary data of Ca^2+^ transient amplitudes in each group. (**E**) Representative M-mode transthoracic views in each group. n = 4–6; mean ± SEM. (**F**, **G**) Summary data of cardiac function (EF, FS). Individual data points are presented in Supplemental Fig. 5B/D/E. *P < 0.05, **P < 0.01, ***P < 0.001 versus sham; ^#^*P* < 0.05, ^##^*P* < 0.01, ^###^*P* < 0.001 versus I/R; ^&^*P* < 0.05, ^&&^*P* < 0.01, ^&&&^*P* < 0.001 versus PD + I/R; ^$$$^*P* < 0.001 versus SERCA2a KO; ^^^*P* < 0.05, ^^^^^*P* < 0.001 versus SERCA2a KO + I/R; ^@^*P* < 0.05, ^@@^*P* < 0.01, ^@@@^*P* < 0.001 versus Ad-GFP + I/R.

T-tubules are essential for effective Ca^2+^-induced Ca^2+^ release and the synchronized contraction of cardiomyocytes. To further verify that SERCA2a regulates T-tubule remodeling by calpain/JPH2, thereby improving the contractile function of cardiomyocytes in I/R, the contraction amplitude of cardiomyocytes was measured under different intervention conditions. As shown (Fig. 5A-B), the contraction amplitude of cardiomyocytes decreased after I/R (*P* < 0.001). After PD pretreatment, the contraction amplitude of cardiomyocytes increased (*P* < 0.01), indicating that calpain affected the contraction amplitude of myocardial cells during myocardial I/R injury. Two weeks after SERCA2a knockout, the contraction amplitude of cardiomyocytes was decreased (*P* < 0.05). The contraction amplitude of cardiomyocytes in the SERCA2a KO + I/R group was lower than the I/R group (*P* < 0.01). SERCA2a overexpression increased cell contraction amplitude in I/R (*P* < 0.01). These results indicated that SERCA2a affected myocardial cell contraction amplitude during I/R. The contraction amplitude of the SERCA2a KO + PD + I/R group was lower than the PD + I/R group (*P* < 0.01), while it was higher than the SERCA2a KO + I/R group (*P* < 0.05). The trend in calcium transients and cardiac functions were consistent with contractility (Fig. 5C–G). These results further indicated that SERCA2a regulates T-tubule remodeling via calpain/JPH2, to improve Ca^2+^ transients, contractility and cardiac function in vivo.

## Discussion

In clinical practice, myocardial I/R injury is a common phenomenon in treating CHD, however its mechanism is very complex. At present, recognized mechanisms include oxygen free radical injury, inflammation, calcium overload and microvascular injury^3^. Among these, Ca^2+^ overload is not only mechanism of myocardial I/R injury, but also the result of reperfusion injury. Ca^2+^ overload leads to mitochondrial dysfunction, over-activation of the Ca^2+^/calmodulin messenger system and apoptotic initiation, which causes I/R injury^30,31^. Therefore, Ca^2+^ overload is a target for mitigating myocardial I/R injury, and has attracted much research attention.

Normal contractile functions in the myocardium depends on normal Ca^2+^ cycles in cardiomyocytes^32^. Normal Ca^2+^ cycling is maintained by calmodulin, SERCA2a, LTCC, RYR2 and NCX (Na^+^-Ca^2+^ exchanger). SERCA2a expression and activity plays vital roles in maintaining Ca^2+^ homeostasis in cardiomyocytes, which are regulated by phospholamban and SUMOylation, etc. In their research, Jiang et al*.* observed that SERCA2a overexpression improved cardiac function in myocardial I/R injury^33^. Our previous studies have shown that decreased SERCA2a activity and expression after myocardial I/R, altered the Ca^2+^ cycle, resulting in Ca^2+^ overload, attenuating cardiac functions^11–13^. In addition, luteolin pretreatment increases SERCA2a expression, activity and stability in myocardial I/R, reducing Ca^2+^ overload and improving myocardial contractile function. However, no studies have verified the role of SERCA2a in maintaining cardiomyocytes, calcium homeostasis and cardiac function in vivo during myocardial I/R, using our experimental approaches.

In our study, we provide evidence that SERCA2a improves cardiomyocyte Ca^2+^ transients, contractility and in vivo cardiac function during myocardial I/R, using SERCA2a knockout mice and SERCA2a overexpression by adenovirus transfection. SERCA2a appears to improve the contractile function of I/R myocardium in two ways: firstly, it reduces Ca^2+^ overload and improves excitation–contraction coupling of the myocardium; and secondly, it decreases mitochondrial membrane potential attenuation caused by Ca^2+^ overload, and reduces apoptosis and cardiomyocyte death. However, the exact mechanism of SERCA2a improving myocardial excitation–contraction coupling in myocardial I/R injury remains poorly understood.

T-tubules are the structural basis of Ca^2+^-induced Ca^2+^ release and excitation–contraction coupling in cardiomyocytes. In HF of different species with different etiologies, T-tubules showed morphological changes or decreased densities, resulting in abnormal Ca^2+^ transients, thereby affecting myocardial systolic function^34,35^. Equally, T-tubule remodeling may occur at the early stages of myocardial damage, rather than being a subsequent modification of heart failure^14^. Some studies have confirmed that T-tubules are plastic, and that some interventions improve T-tubule remodeling, such as SERCA2a gene therapy^15^, beta-adrenergic receptor antagonists^25^, sildenafil^36^, etc. Therefore, T-tubule is a viable target for HF treatment. However, whether the damage of cardiomyocyte contraction amplitude and calcium transient caused by myocardial I/R were also related to T-tubule remodeling and whether SERCA2a was involved in? These are still unknown.

In this study, we found that I/R led to morphological changes in T-tubules, and that SERCA2a ameliorated cardiomyocyte T-tubule remodeling. Thus, our results show that SERCA2a improved myocardial excitation–contraction coupling via T-tubule remodeling in I/R. In a previous study, Lyon et al*.* observed that SERCA2a gene therapy improved Ca^2+^ cycling and excitation–contraction coupling by normalizing T-tubule density in chronic HF, consistent with our previous study^15^. However, our animal model was for myocardial I/R.

Currently, the exact mechanism of T-tubule formation and maintenance is not fully clear. Several studies have shown that JPH2 down-regulation is a key mechanism in T-tubule structural damage^18,19^. JPH2 is down-regulated in several disease models, including HF^26^, dilated or hypertrophic cardiomyopathy^37^ and myocardial I/R^24^.However, it was not clear whether JPH2 affected myocardial contractile amplitude, calcium transients and cardiac function in vivo*,* by regulating T-tubule remodeling in myocardial I/R and whether SERCA2a was involved in. Therefore, we further examined JPH2 expression and localization in cardiomyocytes under different intervention conditions. Guo et al*.* observed that JPH2 was down-regulated by calpain in response to in vitro and in vivo I (20 min)/R (30 min), in agreement with our present study^24^. However, our in vivo I/R model was produced by ligation of LAD for 30 min, followed by reperfusion for 24 h, with PD150606 applied to inhibit in vivo calpain. Our results suggested that SERCA2a regulated T-tubule remodeling through JPH2 in myocardial I/R, and affected myocardial contractile amplitude, calcium transients and cardiac functions in vivo. However, Lyon et al*.* found that SERCA2a gene therapy in mice with HF, could not reverse JPH2 expression. The reason could be that the disease model was different. In our study, JPH2 down-regulation was attenuated by pretreatment with SERCA2a overexpression in I/R. In addition, SERCA2a KO or SERCA2a overexpression in absence of I/R did not influence the expression of JPH2, which suggested SERCA2a expression has a real protecting effect in I/R instead of just displacing the starting point of measured levels. At the same time, we provided information on the safety of using SERCA2a as gene therapy target.

Few studies have focused on JPH2 molecular regulatory mechanisms. At present, two mechanisms are proposed: the first suggests that post-transcriptional regulation is mediated by miR-24^38^, and the second proposes calpain-mediated post-translational regulation^26^. The expression of miR-24 is decreased after myocardial I/R^39,40^. In theory, JPH2 expression should be increased. However, in this study and others, JPH2 expression was down-regulated after myocardial I/R^24^, therefore a miR-24 mediated posttranscriptional regulation is not applicable to myocardial I/R, whereas calpain mediated posttranslational regulation appears to be the main mechanism in reducing JPH2 in myocardial I/R.

Calpain activity increased significantly in several disease models, and then it hydrolyzed several target proteins playing an important role in maintaining cardiac function. It was observed that mitochondrial apoptosis may be reduced, and I/R injury alleviated by inhibiting calpain activity^28^. David et al*.* noted that after 30 min of myocardial ischemia, and 21 days of reperfusion, calpain inhibition reduced scar expansion, ventricular dilatation and dysfunction^41^. In terms of protein topology, there are four calpain splicing sites in JPH2, three at the amino end, and one at the carboxyl end which is the main calpain splicing site^24^. Recently, Wang et al*.* observed that targeting calpain activity in mice with HF, up-regulated JPH2 expression and improved T-tubule remodeling, thereby improving cardiac function^42^.

However, it is not clear whether calpain affects myocardial contractile amplitude, calcium transients, cardiac function in vivo by regulating T-tubule remodeling and whether SERCA2a is involved in I/R. Therefore, we tested changes in calpain activity under different intervention conditions. Our results indicated that SERCA2a regulated contraction amplitude, calcium transients and cardiac function in vivo by calpain/JPH2/T-tubule in I/R. In a previous study, Jang et al*.* found that the calpain inhibitor, MDL28170 attenuated SERCA2a degradation in balloon-injured rat carotid arteries^43^. Previous study found that beta-arrestin2 increased SERCA2a activity^44^. At the same time, calpain could regulate arrestin proteolysis^45^. Similarly, in our study, SERCA2a expression was increased after PD pretreatment, before myocardial I/R injury, suggesting that SERCA2a was also a target of calpain. Calpain possibly regulated SERCA2a by promoting arrestin proteolysis. Equally, there may be interactions between SERCA2a and calpain in myocardial I/R. Calpain activity did not significantly alter after two weeks of SERCA2a KO, suggesting compensatory mechanisms maintain intracellular calcium levels.

There are also some limitations in our present. Firstly, for the limit of the instrument and experimental method, we examine the T-tubule network by isolation of cardiomyocytes instead of in situ confocal imaging of Langendorff-perfused hearts^46^, which could damage the structure of cardiomyocyte. At the same time, it was difficult to obtain T-tubule images in different subepicardial regions in a single intact heart, for example, the infarction zone, border zone, remote zone, and right ventricle of each heart. Secondly, studies are needed to verify the mechanism of SERCA2a regulating calcium balance. Lastly, there may be negative feedback between SERCA2a and calpain, which needs to further study.

**Figure 6:**
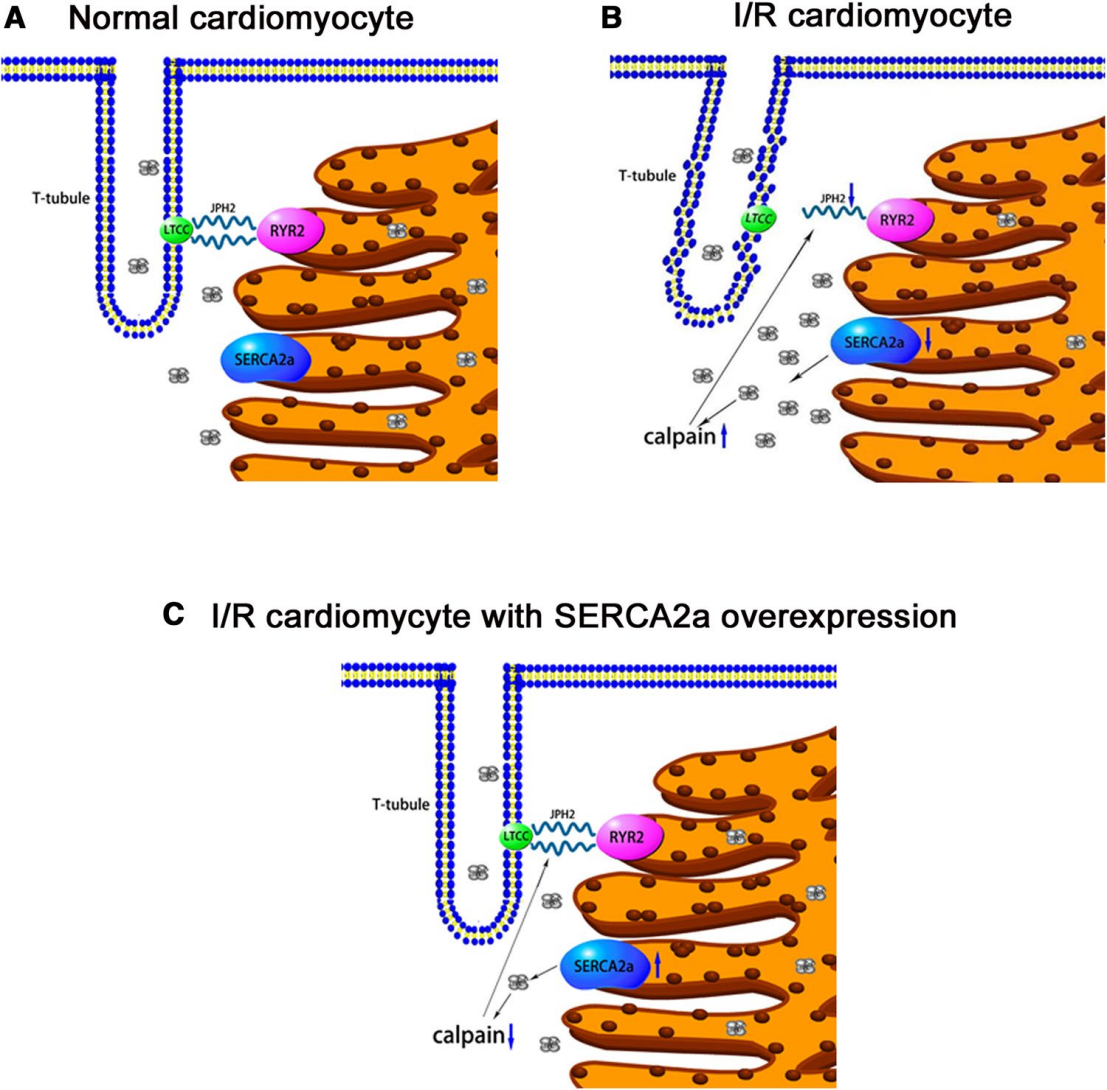
Scheme summarizing the potential mechanism of SERCA2a ameliorating cardiac function via calpain/JPH2/T-tubule in myocardial I/R mice. The image was created using Pathway Builder Tool 2.0(www.proteinlounge.com).

Taken together, this study demonstrated that SERCA2a ameliorated cardiomyocyte T-tubule remodeling in myocardial I/R, thereby improving cardiac function. Mechanistically, SERCA2a ameliorated cardiomyocyte T-tubule remodeling via the calpain/JPH2 pathway to improve cardiac function in myocardial I/R mice (Fig. 6). Furthermore, our study elucidated SERCA2a mediated improvements in cardiac function in I/R, and provided a theoretical and experimental basis for clinical treatments and myocardial I/R injury prevention by targeting SERCA2a.

## Materials and methods

### Animals

All animal experiments conformed to the Guide for the Care and Use of Laboratory Animals (NIH Publication, 8th Edition, 2011) and ARRIVE guidelines. Experimental protocols involving animals were approved by the Animal Ethics Committee of the Xuzhou Medical University, Xuzhou, China. The Serca2^flox/flox^ Tg (αMHC-MerCreMer) mice (Knock Out (KO)) were donated by Professor Ole M. Sejersted (University of Oslo, Oslo, Norway). The mice were administered a tamoxifen (40 mg/kg, intraperitoneal (i.p.) injection, dissolved in peanut oil) to excise the SERCA2a gene from the myocardium^47^. After two weeks, SERCA2a knockout efficiency was verified by western blotting and RT-qPCR. After this, the SERCA2a knockout mice underwent myocardial I/R.

Male C57BL/6j mice, weighing 20–25 g, were randomly divided into sham group, I/R group, PD + I/R group, Ad-GFP + I/R group and Ad-SERCA2a + I/R group. Male Serca2^flox/flox^ Tg (αMHC-MerCreMer) mice, weighing 20–25 g, were randomly divided into con group, SERCA2a KO group, SERCA2a KO + I/R group, SERCA2a KO + PD + I/R group.

### The mouse I/R injury model

The myocardial I/R model was prepared as described previously^48^. Mice were fasting for 12 h before operation. They were anesthetized with 1% sodium pentobarbital (0.1 ml/10 g) by i.p. injection and fixed in a lateral decubitus position on an operating table. The fourth intercostal space was opened to expose the heart, and a silk suture over an inflated balloon was used to ligate the left anterior descending (LAD). After 30 min, the LAD was reperfused by deflating and pulling out the balloon. Dynamic electrocardiogram changes confirmed the model was successfully established. The sham group underwent a similar operative protocol, but without LAD ligation. Subsequent experiments were performed 24 h after myocardial reperfusion. At 30 min before myocardial I/R injury, the mice were i.p. pretreated with PD dissolved in dimethyl sulfoxide (DMSO, Sigma, USA) at 3 mg/kg or equal volume DMSO diluent^28^.

### Adenovirus transfection – SERCA2a overexpression

Adenoviruses (overexpressing SERCA2a) were generated by Hanbio Biotechnology (Shanghai, China). A 10 µL adenovirus aliquot (1.26 × 10^10^ pfu/mL) was injected into the left ventricular myocardium, at three adjacent sites, using a 10 µL microsyringe^49^. After 72 h, adenovirus SERCA2a transfection efficiency was examined by western blotting and RT-qPCR. Hereafter, the adenovirus transfected mice underwent myocardial I/R.

### Isolation of cardiomyocytes

Ca^2+^-free buffer was used to perfuse an excised mouse heart for 4 min, using a Langendorff system. Subsequently, this buffer was switched to an enzyme solution containing Ca^2+^-free buffer, 0.05 mmol/L CaCl_2_, 0.05% collagenase type 2 (Worthington Biochemical corp, USA) and collagenase type 4 (Worthington Biochemical corp, USA). When the heart softened, the left ventricle was cut into small blocks, which were subjected to dispersing. Then, the tissues were filtered and calcium recovery was implemented. Lastly, cells were resuspended in HEPES buffer (mmol L^−1^: 137 NaCl, 1.3 MgSO_4_.7H_2_O, 15 glucose, 5.4 KCl, 1.2 NaH_2_PO_4_, 20 HEPES and 1 CaCl_2_, pH 7.4). The cells were used immediately.

### RT-qPCR

Total RNA was extracted by Trizol Reagent (Invitrogen, Carlsbad, CA, USA). SYBR Green (Tian Gen, Shanghai, China) was used for RT-qPCR, as previously described^50^. The relative expression levels of SERCA2a were normalized to GAPDH. The primers were:

SERCA2a Forward: 5′-CGGTGCCTTTGTTGTCTCCA -3’.

Reverse: 5′-ACCTGACTTTCGTCGGCTGTGT -3’.

GAPDH Forward: 5′- GGCCTCCAAGGAGTAAGAAA -3’.

Reverse: 5′- GCCCCTCCTGTTATTATGG -3’.

### Western blot analysis

Protein samples were extracted from myocardial tissue using a lysis solution (Beyotime Biotechnology, China). The samples were resolved by SDS–polyacrylamide gel electrophoresis, and transferred to polyvinylidene fluoride membranes (Merck Millipore, Billerica, MA, USA). All the blots were cut prior to hybridization with antibodies. Primary antibodies against SERCA2a (1:40,000) (Abcam, Cambridge, UK), α-fodrin (1:1000) (Abcam, Cambridge, UK), JPH2 (1:1000) ( Santa Cruz Biotechnology, Santa Cruz, CA, USA) and GAPDH (1:5000) ( Proteintech Group , China)were immuno-blotted overnight at 4℃. After this, membranes were immuno-blotted at room temperature for 1 h with corresponding secondary antibodies (1:5000) (ZSGB-BIO, Beijing, China). A chemiluminescence detection system (Tanon Imaging System, Tanon, Shanghai, China) was used to visualize protein bands. The data were analyzed on ImageJ 1.51 (NIH, USA).

### Calpain activity assay

The Calpain Activity Assay kit (BioVison, Milpitas, CA, USA) was used to detect calpain activity. Myocardial tissue was cut into pieces and homogenized in 100 µL extraction buffer. Samples were then lysed on ice for 20 min, and centrifuged for 1 min (10000 g, 4 °C). Then, 100 µg protein was mixed with calpain substrate and reaction buffer, and balanced with extraction buffer. Fluorescence was measured at 37 °C for 1 h using a microplate fluorescence reader (excitation wavelength of 400 nm and emission wavelength of 505 nm). Calpain activity was expressed as relative fluorescence. We also assessed the expression levels of the 150 kDa fodrin breakdown product (FBDP) by western blotting, also to reflect calpain activity^28^.

### T-tubule visualization and immunofluorescence microscopy

To visualize T-tubules, isolated cardiomyocytes were incubated with 5 uM MM 4–64 (AAT Bioquest Inc, CA, USA) in HEPES buffer for 30 min, after which they were washed in HEPES buffer for 10 min. The Olympus FV10i confocal microscope (Olympus, Japan) was used to acquire images (excitation wavelength 558 nm and emission wavelength 734 nm). Images were processed using an Olympus FluoView FV10 (Version 4.2). TTpower was analyzed by imageJ as previously described^51^.

For immunofluorescence microscopy, cardiomyocytes were fixed at room temperature for 10 min in 4% polyformaldehyde, permeabilized in 0.2% Triton X-100 for 10 min and blocked with blocking buffer (1% BSA, 22.5 mg/ml glycine, 1 × PBS, 0.1% Tween 20). Cardiomyocytes were then incubated with primary antibodies against JPH2 (1:200) (Santa Cruz Biotechnology, Santa Cruz, CA, USA) and RYR2 (1:200) (Proteintech Group, China) overnight at 4 °C, and the next day, with Alexa Fluor-labeled secondary antibodies at room temperature for 1 h. Finally, cardiomyocytes were mounted using fluorescent mounting medium and images acquired using the Olympus FV10i confocal microscope. TTpower JPH2 and Pearson correlation coefficient (JPH2 and RYR2) were analyzed by imageJ, as previously described^51^.

### *Cardiomyocyte contractility and Ca*^2+^*transient measurements*

The IonOptix Myocam system (IonOptix Corp., Milton, MA, USA) assessed cardiomyocyte contractile properties. Ca^2+^ transients in cardiomyocytes loaded with Fura-2 AM (Dojindo Labratories, Japan, excitation wavelength 340/380 nm and emission wavelength of 510 nm) were measured as previously described^52,53^. The percentage of Fura-2 fluorescence intensity change (ΔFFI) to resting cell Fura-2 fluorescence intensity (FFI_Diastolic_) reflected Ca^2+^ transient amplitude. Fluorescence ratios (R = F340/F380) were used to calculate cytosolic Ca^2+^ concentrations: [Ca^2+^]_i_ = Kd × (R-Rmin) / (Rmax-R) × Sf2 / Sb2. R_min_ and R_max_ are fluorescence ratios under calcium deficiency and saturation conditions, respectively. Sf2 and Sb2 are fluorescence values (excitation at 380 nm) under calcium deficiency and saturation conditions, respectively. K_d_ is the dissociation constant for Fura-2-calcium binding (225 nM).

### Echocardiography

Echocardiography was performed according to a previous protocol^54^. Mice were examined by transthoracic echocardiography using a Vevo1100 high resolution ultrasound imaging system (Visual Sonics, Canada), the probe being MS-400 with a frequency of 30 MHz. M-mode echocardiography detected related indicators, including ejection fraction (EF) and fractional shortening (FS). All indices were averaged over three consecutive cardiac cycles.

### Statistical analysis

Data are shown as the mean ± standard error of the mean, and all data were analyzed on GraphPad Prism 5.0 software. We used two-tailed Student's t-tests for the differences of mRNA and protein expression between con group and SERCA2a KO group, and used one-way ANOVA, followed by a Bonferroni post hoc correction tests for all group comparisons. A *P* < 0.05 value was considered statistically significant. All experiments were performed at least three times.

## Supplementary Information

Supplementary Figures
