# Supplementary material for: SERCA2a ameliorates cardiomyocyte T-tubule remodeling via the calpain/JPH2 pathway to improve cardiac function in myocardial ischemia/reperfusion mice
Source: Sci Rep. 2021 Jan 21;11:2037. doi: 10.1038/s41598-021-81570-4 (PMC7820433; doi:10.1038/s41598-021-81570-4)
Supplement: Supplementary file 1 — Supplementary Figures [file 41598_2021_81570_MOESM1_ESM.pdf]

# SERCA2a ameliorates cardiomyocyte T-tubule remodeling via the calpain/JPH2 pathway to improve cardiac function in myocardial ischemia/reperfusion mice

Shuai Wang[a], You Zhou[a], Yuanyuan Luo[b], Rongsheng Kan[a], Jingwen Chen[a], Haochen Xuan[b], Chaofan Wang[b], Junhong Chen[b], Tongda Xu[b*], Dongye Li[a,b*]

[a]Institute of Cardiovascular Disease Research, Xuzhou Medical University, 221002, Xuzhou, Jiangsu, PR China

[b]Department of Cardiology, The Affiliated Hospital of Xuzhou Medical University, 221006, Xuzhou, Jiangsu, PR China

The first three authors contributed equally for this paper.

[*]Correspondence to: Dongye Li or Tongda Xu

Dongye Li, Institute of Cardiovascular Disease Research, Xuzhou Medical University, 84 West Huaihai Road, 221002 Xuzhou, Jiangsu, PR China.

Department of Cardiology, the Affiliated Hospital of Xuzhou Medical University,99 West Huaihai Road, 221006, Xuzhou, Jiangsu, PR China.

Tel: +86-516-85582763;

Fax: +86-516-85582753.

E-Mail: dongyeli@xzhmu.edu.cn

Tongda Xu, Department of Cardiology, The Affiliated Hospital of Xuzhou Medical University, 99 West Huaihai Road, 221006, Xuzhou, Jiangsu, PR China.

E-mail: xutongda3004@163.com.

The original blots of each figure:

Supplementary Figure 1D:

SERCA2a:

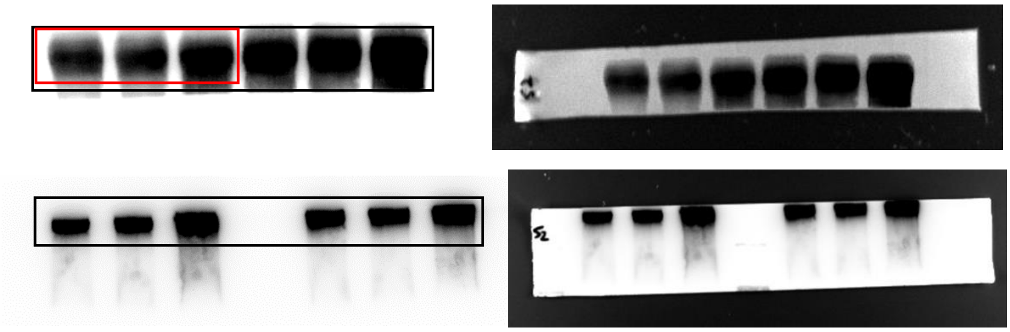

The different exposure blots:

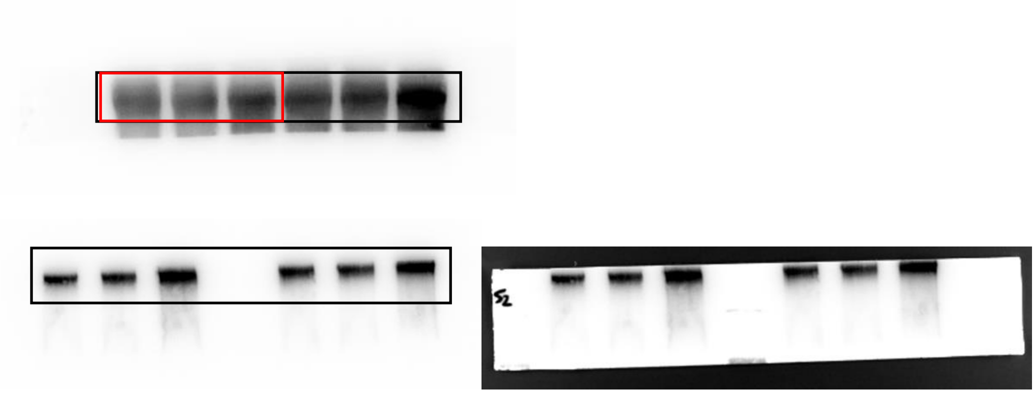

GAPDH:

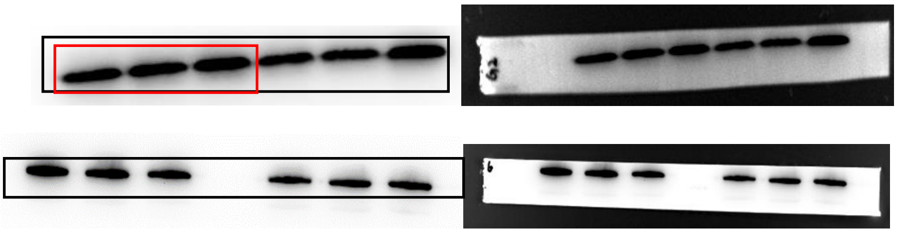

Supplementary Figure 1F:

SERCA2a:

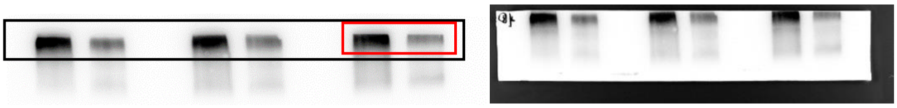

GAPDH:

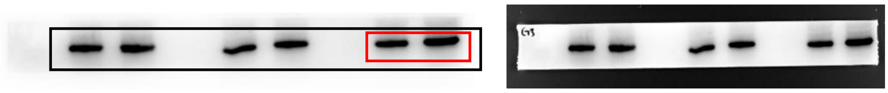

Supplementary Figure 2A:

SERCA2a:

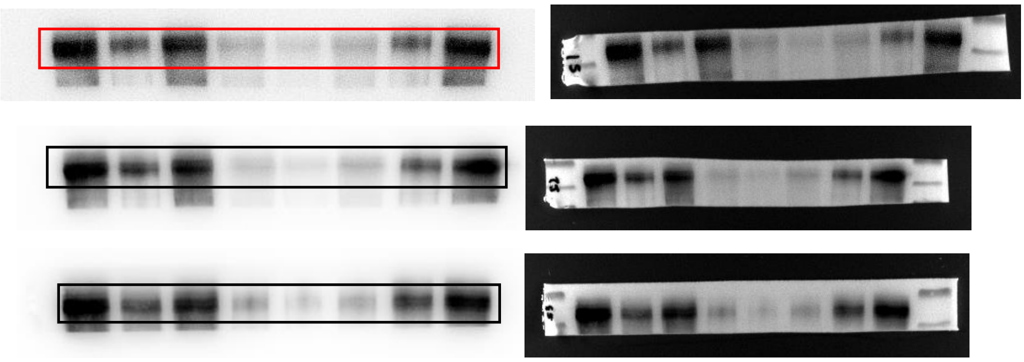

GAPDH:

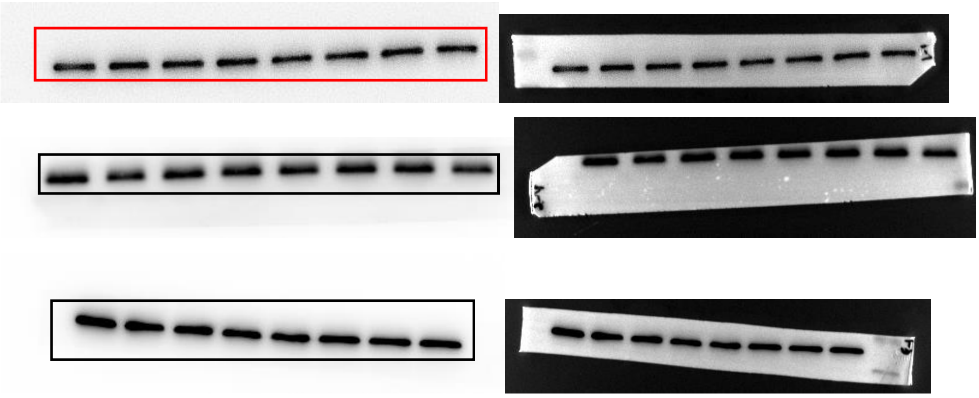

Supplementary Figure 2C:

FBDP:

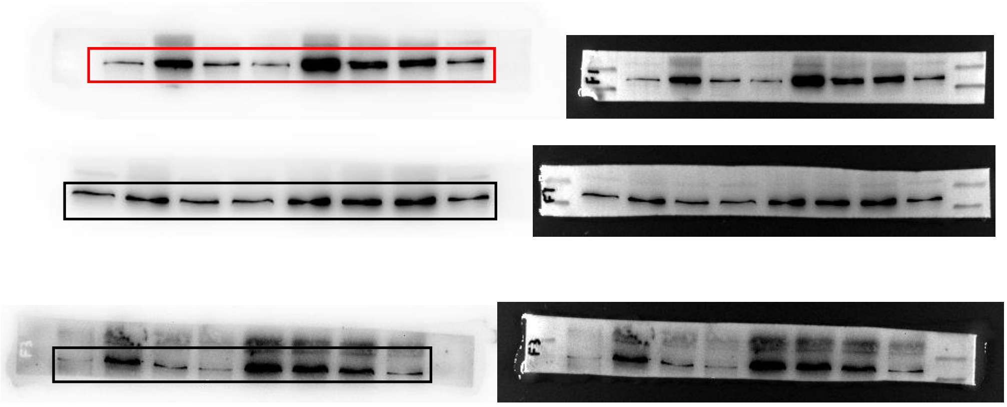

GAPDH:

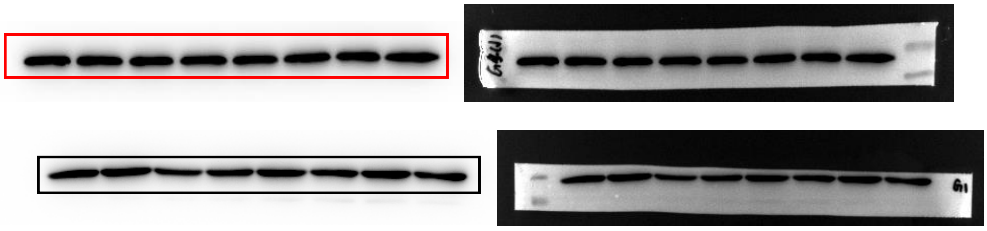

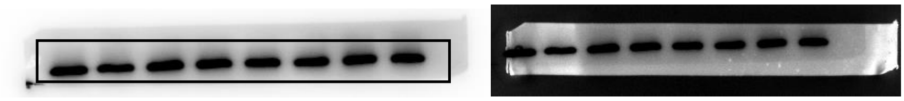

Supplementary Figure 3D:

JPH2:

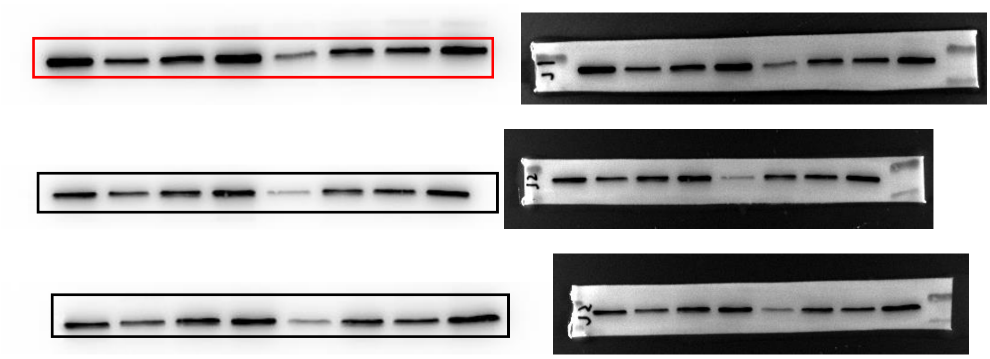

GAPDH:

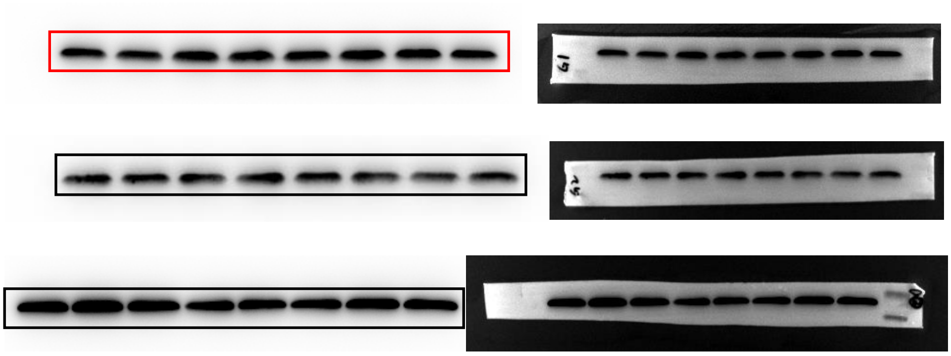

Individual data points figure:

Supplementary Figure 1C-F:

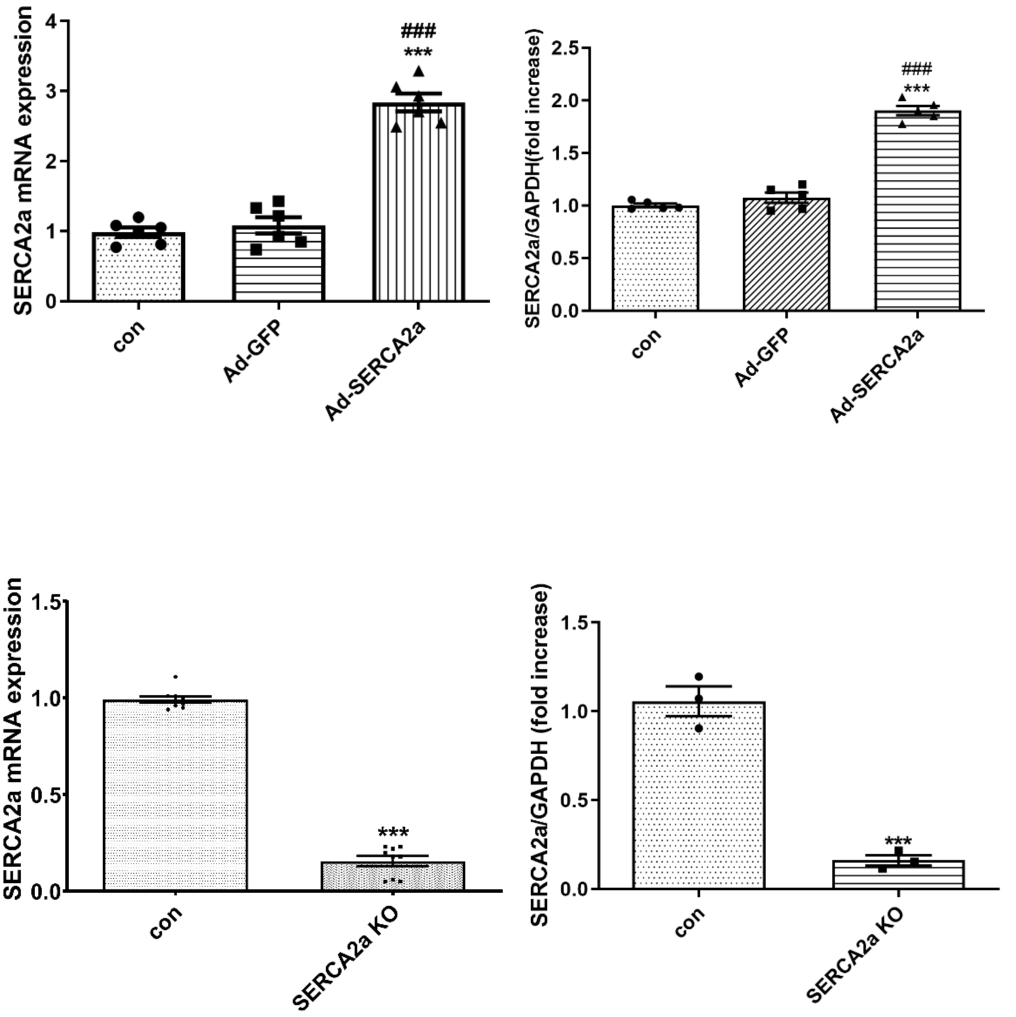


Supplementary Figure 2A-D:

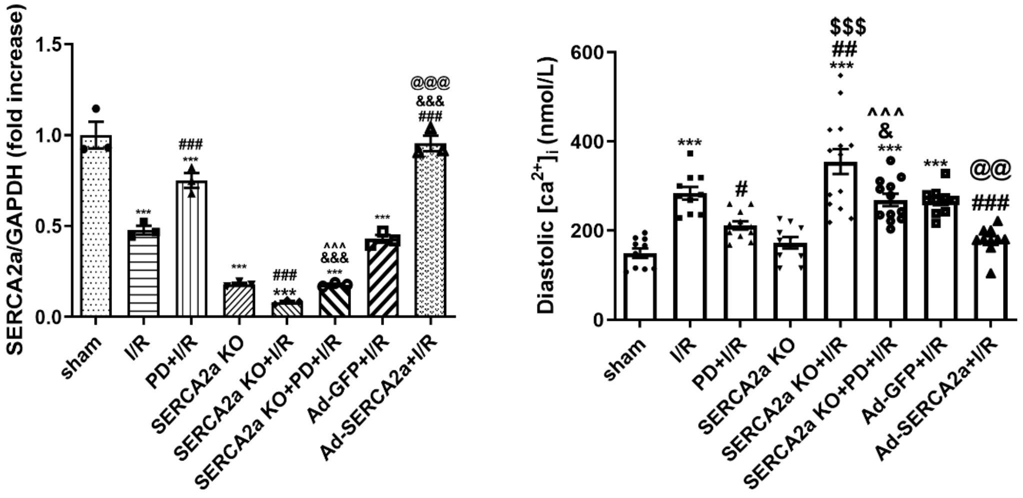

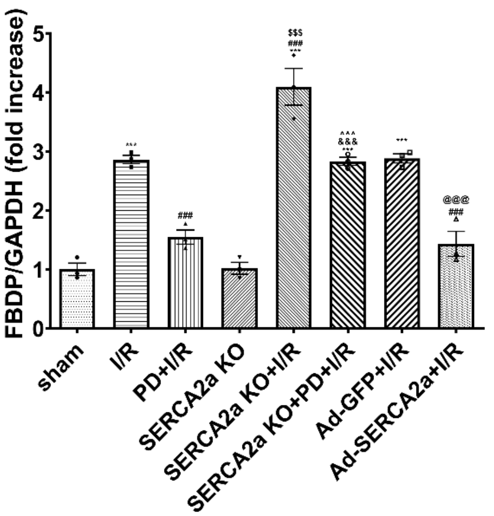


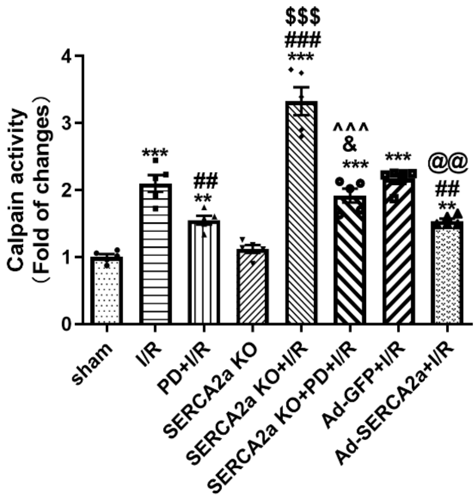


Supplementary Figure 3B-D：

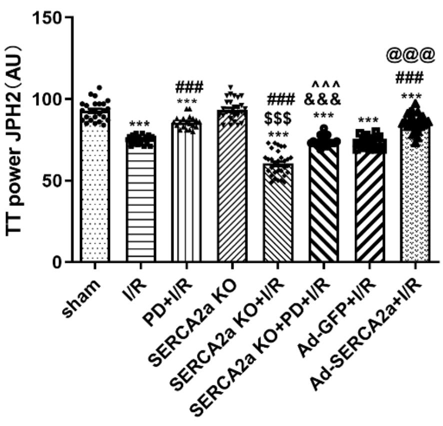


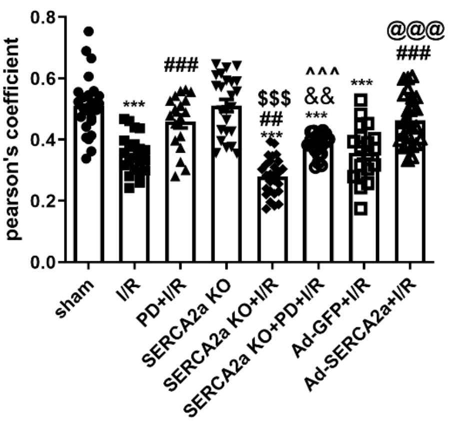


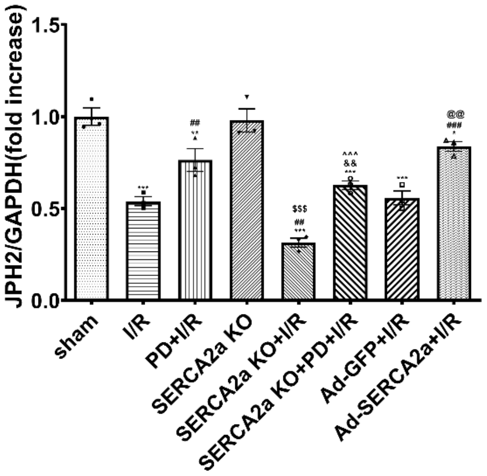

Supplementary Figure 4B：

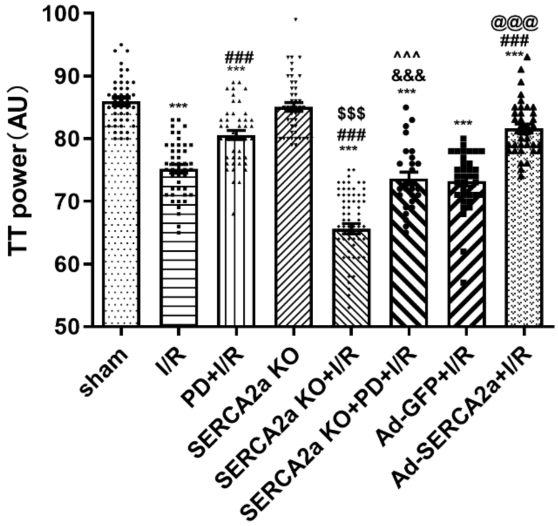


Supplementary Figure 5B/D/E：

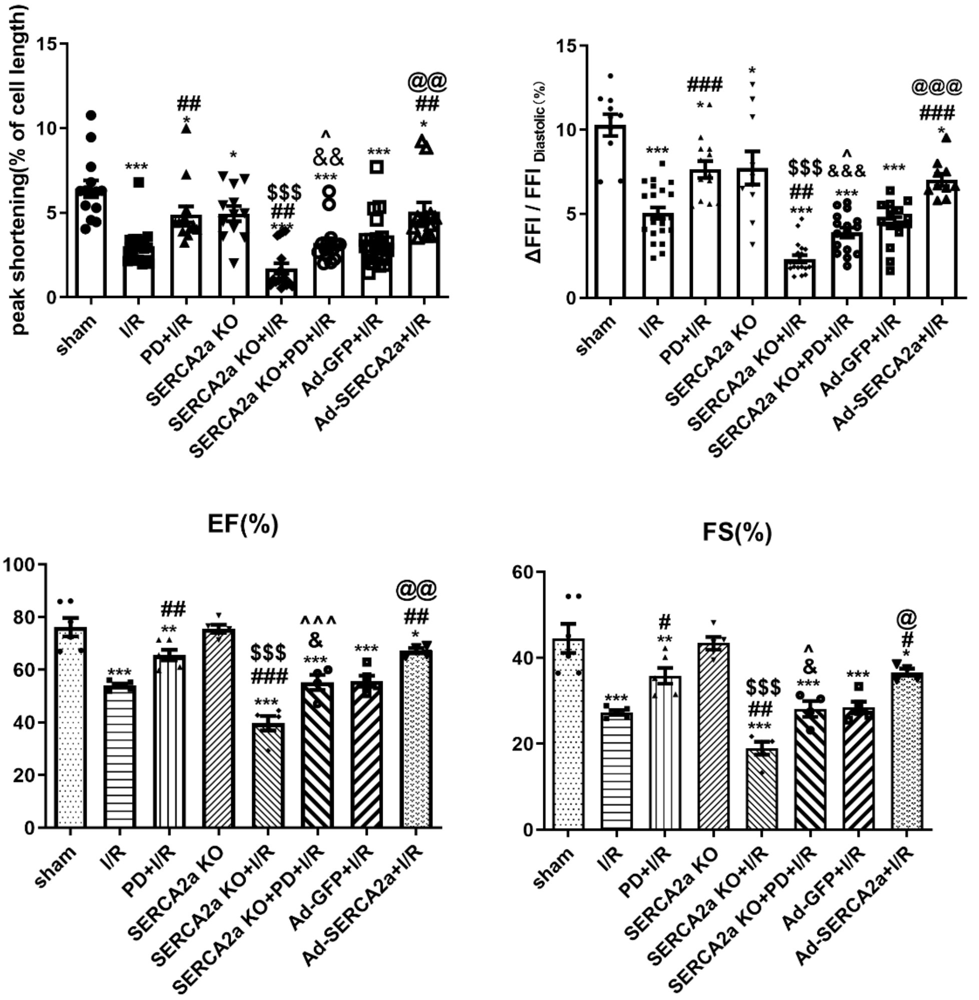

Figure S1:

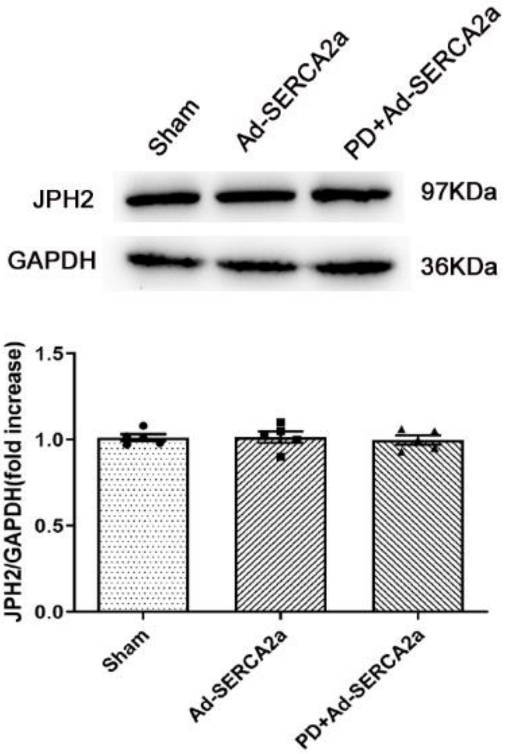


The level of SERCA2a protein expression in each group; n=5; mean±SEM. GAPDH was used as the internal control. The grouping of gels/blots cropped from different parts of the same gel. Uncropped blots are available below.

The original blots of Figure S1:

JPH2:

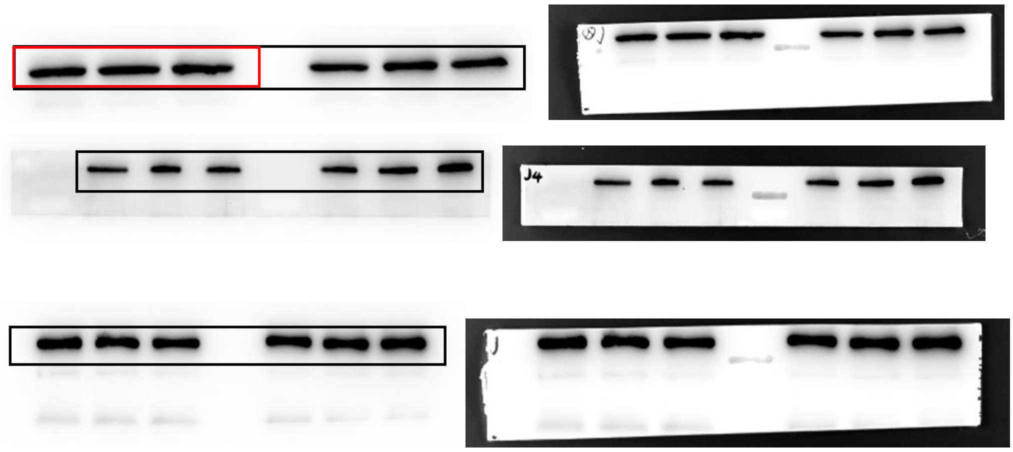

GAPDH:

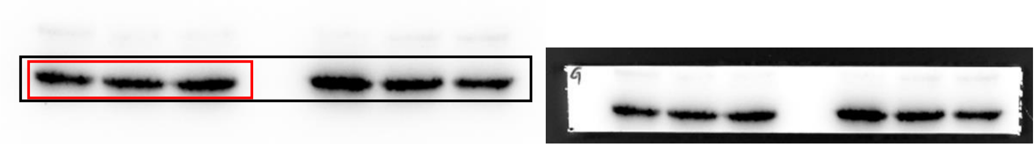

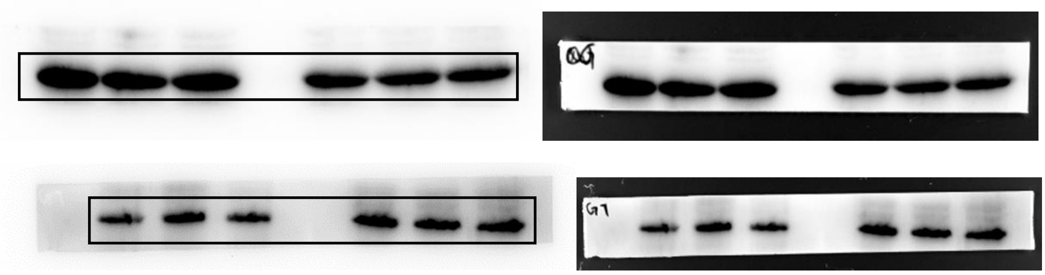
G7
